# [2-(Diphenyl­phosphan­yl)benzene­thiol­ato-κ^2^ *P*,*S*](pyridine-2-thiol­ato-κ*S*)(triphenyl­phosphine-κ*P*)palladium(II)

**DOI:** 10.1107/S160053681003357X

**Published:** 2010-08-28

**Authors:** Raúl Ríos-Sanchez, Simón Hernández-Ortega, David Morales-Morales, Alcives Avila-Sorrosa

**Affiliations:** a Instituto de Química, Universidad Nacional Autónoma de México, Circuito Exterior, Ciudad Universitaria, México 04510, Mexico

## Abstract

In the title compound, [Pd(C_5_H_4_NS)(C_18_H_14_PS)(C_18_H_15_P)], the Pd^II^ atom has a slightly distorted square-planar environment. Two coordination sites are occupied by a *P*,*S*-chelating 2-(diphenyl­phosphan­yl)benzene­thiol­ate ligand and the other two by a P atom from a triphenyl­phosphine ligand and an S atom from a pyridine-2-thiol­ate ligand, exhibiting a *trans* arrangement of the two P-donor atoms. In the crystal structure, weak intra- and inter­molecular C—H⋯π and π–π inter­actions are observed. The pyridyl ring is equally disordered over two positions.

## Related literature

For general background to non-symmetric chelating ligands, see: Braunstein & Naud (2001 ▶); Dilworth *et al.* (2000 ▶); Dilworth & Weatley (2000 ▶); Serrano-Becerra *et al.* (2010 ▶); Solano-Prado *et al.* (2010 ▶). For a related structure, see: Benefiel *et al.* (1984 ▶). For the synthesis of transition metal complexes with *P*,*S*-non-symmetric ligands, see: Canseco-González *et al.* (2003 ▶, 2004 ▶); Fierro-Arias *et al.* (2008 ▶); Gómez-Benítez *et al.* (2003 ▶, 2007*a* ▶,*b* ▶); Hernández-Ortega & Morales-Morales (2008 ▶); Morales-Morales *et al.* (2002*a* ▶,*b* ▶); Ríos-Moreno *et al.* (2005 ▶).
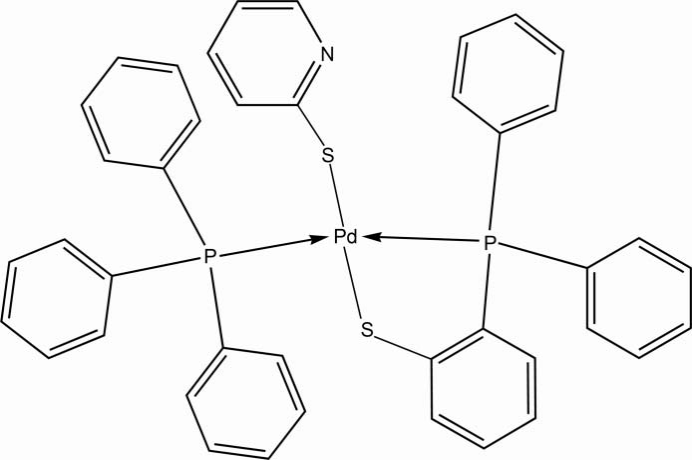


## Experimental

### 

#### Crystal data

[Pd(C_5_H_4_NS)(C_18_H_14_PS)(C_18_H_15_P)]
*M* *_r_* = 772.14
Triclinic, 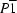

*a* = 11.399 (5) Å
*b* = 12.178 (5) Å
*c* = 13.467 (5) Å
α = 83.623 (5)°
β = 76.379 (5)°
γ = 75.135 (5)°
*V* = 1753.5 (12) Å^3^
*Z* = 2
Mo *K*α radiation
μ = 0.77 mm^−1^
*T* = 298 K
0.34 × 0.06 × 0.05 mm

#### Data collection

Bruker SMART APEX CCD diffractometer
Absorption correction: multi-scan (*SADABS*; Bruker, 2001 ▶) *T*_min_ = 0.780, *T*_max_ = 0.963
14855 measured reflections
6429 independent reflections
4588 reflections with *I* > 2σ(*I*)
*R*_int_ = 0.046

#### Refinement

*R*[*F* ^2^ > 2σ(*F* ^2^)] = 0.053
*wR*(*F* ^2^) = 0.123
*S* = 1.02
6429 reflections
470 parameters
186 restraints
H-atom parameters constrained
Δρ_max_ = 0.71 e Å^−3^
Δρ_min_ = −0.30 e Å^−3^

### 

Data collection: *SMART* (Bruker, 2007 ▶); cell refinement: *SAINT-Plus* (Bruker, 2007 ▶); data reduction: *SAINT-Plus*; program(s) used to solve structure: *SHELXS97* (Sheldrick, 2008 ▶); program(s) used to refine structure: *SHELXL97* (Sheldrick, 2008 ▶); molecular graphics: *SHELXTL* (Sheldrick, 2008 ▶); software used to prepare material for publication: *SHELXTL*.

## Supplementary Material

Crystal structure: contains datablocks I, global. DOI: 10.1107/S160053681003357X/hy2339sup1.cif

Structure factors: contains datablocks I. DOI: 10.1107/S160053681003357X/hy2339Isup2.hkl

Additional supplementary materials:  crystallographic information; 3D view; checkCIF report

## Figures and Tables

**Table 1:** Selected bond lengths (Å)

Pd1—P1	2.2585 (15)
Pd1—P2	2.3575 (15)
Pd1—S1	2.2999 (15)
Pd1—S2	2.3374 (15)

**Table 2:** Intra- and inter­molecular C—H⋯π and π–π inter­actions (Å)

H/centroid	centroid	distance
N37,C38–C42	C19–C24	3.93 (2)
N37*A*,C38,C39*A*–C42*A*	C7–C12	4.00 (2)
C31–C36	C31^i^–C36^i^	3.76 (2)
H24	C25–C30	3.17
H30	C31–C36	3.11
H36	C19–C24	3.20
H29	C31^ii^–C36^ii^	3.06

Symmetry codes: (i) −*x*, −*y* + 1, −*z* + 1; (ii) −*x* + 1, −*y* + 1, −*z* + 1.

**Fig. 1.**
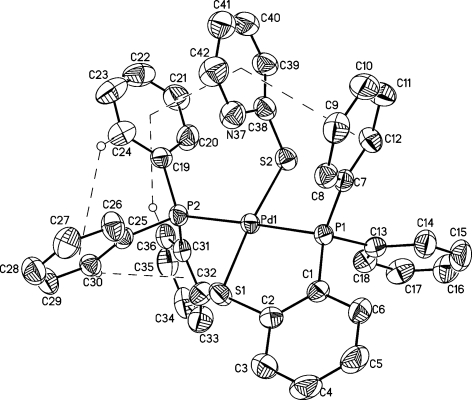
The molecular structure of the title compound. Displacement ellipsoids are drawn at the 40% probability level. Only the H atoms involved in C—H···π interactions are shown. Dashed lines denote C—H···π and π–π interactions. The second parts of the disordered atoms have been omitted for clarity.

## supplementary crystallographic information

### Comment

Nonsymmetric chelating ligands have had a renaissance in recent years due to the potential properties they may confer to the compounds they form (Dilworth & Weatley, 2000). Thus, steric and electronic effects can be modified easily due in part to the pronounced chemical differences among the donor atoms in these ligands (Serrano-Becerra *et al.*, 2010; Solano-Prado *et al.*, 2010). Properties such as hemilability (Braunstein & Naud, 2001) have been invoked to explain the often observed enhanced reactivities of these metallic complexes, turning these species very attractive for their potential applications in homogeneous catalysis and metal mediated organic synthesis (Dilworth *et al.*, 2000). Thus, given our continuous interest in the synthesis of transition metal complexes bearing P,S-nonsymmetric hybrid ligands (Canseco-González *et al.*, 2003, 2004; Fierro-Arias *et al.*, 2008; Gómez-Benítez *et al.*, 2003, 2007*a*,b; Hernández-Ortega & Morales-Morales, 2008; Morales-Morales *et al.*, 2002*a*,b; Ríos-Moreno *et al.*, 2005), we report here the structure of the title complex.

The title palladium complex (Fig. 1) consists of a diphenylphosphinobenzenethiolate ligand coordinated in a bidentated manner, a triphenylphosphine ligand arranged in a *trans* configuration with respect to the P atom of the P,S-chelating ligand and a pyridin-2-thiolate ligand coordinated by the S atom. The Pd—S distances observed are different (0.04 Å) (Table 1), while the Pd—P distance in the triphenylphosphine ligand are slightly longer than that observed in the diphenylphosphine fragment of the P,S-ligand (0.1 Å), due to the preferred bite angle of the chelate ligand. The disordered pyridyl ring of the pyridin-2-thiolate ligand and the phenyl rings (C7–C12 and C19–C24) exhibits important intramolecular face–to–face π–π interactions [centroid–centroid distances = 4.00 (2) and 3.93 (2) Å]. Additionally, the crystal packing is also supported by intermolecular C—H···π and π–π interactions (Table 2).

### Experimental

The title compound was synthesized from the metathetical reaction of triphenylphosphine-2-(diphenylphosphine)benzenethiolate palladium(II) chloride [κ^2^-(SC_6_H_4_-2-PPh_2_)Pd(PPh_3_)Cl] (100 mg, 143 mmol) with lead 2-mercaptopirydine [Pb(C_5_H_4_N-2-S)_2_] (31 mg, 72 mmol) in a 2:1 molar ratio. The title compound was obtained as a bright yellow microcrystalline powder in a 92% (101 mg) yield.

### Refinement

H atoms were positioned geometrically and refined as riding atoms, with C—H = 0.93Å and with *U*_iso_(H) = 1.2*U*_eq_(C). The pyridyl ring is disordered and was refined in two positions each with an occupancy factor of 0.50 (2).

### Crystal data

**Table:**

[Pd(C_5_H_4_NS)(C_18_H_14_PS)(C_18_H_15_P)]	*Z* = 2
*M**_r_* = 772.14	*F*(000) = 788
Triclinic, *P*1	*D*_x_ = 1.462 Mg m^−^^3^
Hall symbol: -P 1	Mo *K*α radiation, λ = 0.71069 Å
*a* = 11.399 (5) Å	Cell parameters from 3920 reflections
*b* = 12.178 (5) Å	θ = 2.2–24.6°
*c* = 13.467 (5) Å	µ = 0.77 mm^−^^1^
α = 83.623 (5)°	*T* = 298 K
β = 76.379 (5)°	Prism, yellow
γ = 75.135 (5)°	0.34 × 0.06 × 0.05 mm
*V* = 1753.5 (12) Å^3^	

### Data collection

**Table:**

Bruker SMART APEX CCD diffractometer	6429 independent reflections
Radiation source: fine-focus sealed tube	4588 reflections with *I* > 2σ(*I*)
graphite	*R*_int_ = 0.046
φ and ω scans	θ_max_ = 25.4°, θ_min_ = 1.6°
Absorption correction: multi-scan (*SADABS*; Bruker, 2001)	*h* = −13→13
*T*_min_ = 0.780, *T*_max_ = 0.963	*k* = −14→14
14855 measured reflections	*l* = −16→16

### Refinement

**Table:**

Refinement on *F*^2^	Primary atom site location: structure-invariant direct methods
Least-squares matrix: full	Secondary atom site location: difference Fourier map
*R*[*F*^2^ > 2σ(*F*^2^)] = 0.053	Hydrogen site location: inferred from neighbouring sites
*wR*(*F*^2^) = 0.123	H-atom parameters constrained
*S* = 1.02	*w* = 1/[σ^2^(*F*_o_^2^) + (0.0518*P*)^2^ + 0.5024*P*] where *P* = (*F*_o_^2^ + 2*F*_c_^2^)/3
6429 reflections	(Δ/σ)_max_ = 0.038
470 parameters	Δρ_max_ = 0.71 e Å^−^^3^
186 restraints	Δρ_min_ = −0.30 e Å^−^^3^

### Fractional atomic coordinates and isotropic or equivalent isotropic displacement parameters (Å^2^)

**Table:**

	*x*	*y*	*z*	*U*_iso_*/*U*_eq_	Occ. (<1)
Pd1	0.26508 (3)	0.20691 (3)	0.25871 (3)	0.03896 (13)	
S1	0.41538 (12)	0.10047 (11)	0.34128 (10)	0.0527 (3)	
S2	0.10300 (12)	0.29994 (11)	0.17836 (10)	0.0513 (3)	
P1	0.21862 (11)	0.04240 (10)	0.23856 (9)	0.0383 (3)	
P2	0.31291 (12)	0.37713 (11)	0.28524 (10)	0.0435 (3)	
C1	0.3252 (4)	−0.0722 (4)	0.2934 (3)	0.0416 (11)	
C2	0.4067 (4)	−0.0419 (4)	0.3403 (4)	0.0442 (12)	
C3	0.4853 (5)	−0.1262 (5)	0.3891 (4)	0.0609 (15)	
H3	0.5392	−0.1068	0.4225	0.073*	
C4	0.4824 (6)	−0.2371 (5)	0.3874 (5)	0.0697 (17)	
H4	0.5348	−0.2930	0.4203	0.084*	
C5	0.4042 (5)	−0.2682 (5)	0.3384 (5)	0.0681 (16)	
H5	0.4050	−0.3446	0.3370	0.082*	
C6	0.3249 (5)	−0.1864 (4)	0.2916 (4)	0.0546 (13)	
H6	0.2711	−0.2069	0.2588	0.065*	
C7	0.2388 (4)	0.0109 (4)	0.1058 (3)	0.0400 (11)	
C8	0.3553 (5)	−0.0422 (4)	0.0520 (4)	0.0492 (12)	
H8	0.4197	−0.0698	0.0864	0.059*	
C9	0.3772 (5)	−0.0547 (5)	−0.0516 (4)	0.0603 (15)	
H9	0.4561	−0.0902	−0.0868	0.072*	
C10	0.2838 (7)	−0.0154 (5)	−0.1027 (4)	0.0710 (17)	
H10	0.2990	−0.0233	−0.1727	0.085*	
C11	0.1676 (6)	0.0357 (5)	−0.0511 (4)	0.0708 (17)	
H11	0.1033	0.0606	−0.0859	0.085*	
C12	0.1447 (5)	0.0509 (4)	0.0533 (4)	0.0529 (13)	
H12	0.0660	0.0880	0.0876	0.063*	
C13	0.0628 (4)	0.0290 (4)	0.3020 (3)	0.0449 (12)	
C14	0.0115 (5)	−0.0538 (5)	0.2790 (4)	0.0682 (16)	
H14	0.0556	−0.1031	0.2276	0.082*	
C15	−0.1055 (6)	−0.0635 (6)	0.3322 (5)	0.083 (2)	
H15	−0.1394	−0.1194	0.3163	0.100*	
C16	−0.1711 (6)	0.0075 (6)	0.4073 (5)	0.0814 (19)	
H16	−0.2494	0.0001	0.4428	0.098*	
C17	−0.1224 (5)	0.0892 (5)	0.4307 (5)	0.0714 (17)	
H17	−0.1675	0.1381	0.4820	0.086*	
C18	−0.0057 (5)	0.0999 (4)	0.3782 (4)	0.0556 (13)	
H18	0.0270	0.1562	0.3949	0.067*	
C19	0.2835 (5)	0.5004 (4)	0.1960 (4)	0.0511 (13)	
C20	0.1634 (6)	0.5559 (5)	0.1907 (5)	0.0710 (17)	
H20	0.0985	0.5280	0.2320	0.085*	
C21	0.1352 (7)	0.6508 (5)	0.1270 (6)	0.084 (2)	
H21	0.0530	0.6878	0.1268	0.101*	
C22	0.2296 (8)	0.6888 (6)	0.0650 (5)	0.089 (2)	
H22	0.2124	0.7535	0.0222	0.107*	
C23	0.3497 (8)	0.6337 (6)	0.0642 (5)	0.093 (2)	
H23	0.4136	0.6594	0.0190	0.111*	
C24	0.3780 (6)	0.5397 (5)	0.1301 (4)	0.0683 (16)	
H24	0.4604	0.5033	0.1298	0.082*	
C25	0.4744 (4)	0.3612 (4)	0.2925 (4)	0.0440 (11)	
C26	0.5669 (5)	0.2975 (5)	0.2206 (4)	0.0632 (15)	
H26	0.5461	0.2621	0.1717	0.076*	
C27	0.6903 (6)	0.2867 (5)	0.2217 (5)	0.0710 (17)	
H27	0.7521	0.2447	0.1726	0.085*	
C28	0.7229 (5)	0.3366 (5)	0.2934 (5)	0.0685 (16)	
H28	0.8062	0.3283	0.2939	0.082*	
C29	0.6308 (5)	0.3994 (5)	0.3650 (4)	0.0653 (15)	
H29	0.6521	0.4344	0.4139	0.078*	
C30	0.5090 (5)	0.4110 (4)	0.3649 (4)	0.0490 (12)	
H30	0.4480	0.4530	0.4145	0.059*	
C31	0.2270 (4)	0.4299 (4)	0.4100 (4)	0.0460 (12)	
C32	0.2156 (5)	0.3516 (5)	0.4923 (4)	0.0588 (14)	
H32	0.2460	0.2742	0.4824	0.071*	
C33	0.1589 (6)	0.3888 (6)	0.5892 (5)	0.0779 (19)	
H33	0.1539	0.3362	0.6447	0.093*	
C34	0.1106 (5)	0.5011 (7)	0.6040 (5)	0.078 (2)	
H34	0.0713	0.5250	0.6694	0.093*	
C35	0.1194 (5)	0.5791 (6)	0.5236 (6)	0.0740 (18)	
H35	0.0855	0.6560	0.5339	0.089*	
C36	0.1780 (5)	0.5440 (5)	0.4277 (4)	0.0572 (14)	
H36	0.1852	0.5978	0.3733	0.069*	
C38	0.1818 (5)	0.3228 (4)	0.0538 (4)	0.0555 (12)	
N37	0.3081 (7)	0.2875 (18)	0.0324 (10)	0.068 (3)	0.50 (2)
C39	0.1267 (10)	0.4052 (14)	−0.0117 (8)	0.064 (3)	0.50 (2)
H39	0.0448	0.4452	0.0101	0.077*	0.50 (2)
C40	0.1895 (14)	0.4282 (14)	−0.1058 (8)	0.071 (3)	0.50 (2)
H40	0.1499	0.4812	−0.1499	0.086*	0.50 (2)
C41	0.3102 (14)	0.3744 (16)	−0.1364 (9)	0.076 (3)	0.50 (2)
H41	0.3525	0.3819	−0.2040	0.092*	0.50 (2)
C42	0.3674 (10)	0.3094 (16)	−0.0654 (11)	0.077 (3)	0.50 (2)
H42	0.4523	0.2777	−0.0845	0.092*	0.50 (2)
N37A	0.3023 (8)	0.2694 (18)	0.0152 (9)	0.067 (3)	0.50 (2)
C39A	0.1072 (10)	0.3708 (15)	−0.0149 (7)	0.064 (3)	0.50 (2)
H39A	0.0213	0.3906	0.0082	0.077*	0.50 (2)
C40A	0.1566 (14)	0.3893 (16)	−0.1138 (7)	0.073 (3)	0.50 (2)
H40A	0.1053	0.4265	−0.1577	0.088*	0.50 (2)
C41A	0.2798 (14)	0.3544 (16)	−0.1503 (8)	0.077 (3)	0.50 (2)
H41A	0.3165	0.3743	−0.2169	0.092*	0.50 (2)
C42A	0.3480 (11)	0.2893 (16)	−0.0863 (9)	0.072 (3)	0.50 (2)
H42A	0.4311	0.2562	−0.1131	0.086*	0.50 (2)

### Atomic displacement parameters (Å^2^)

**Table:**

	*U*^11^	*U*^22^	*U*^33^	*U*^12^	*U*^13^	*U*^23^
Pd1	0.0445 (2)	0.0354 (2)	0.0403 (2)	−0.01175 (16)	−0.01242 (16)	−0.00360 (15)
S1	0.0589 (8)	0.0490 (8)	0.0591 (8)	−0.0123 (7)	−0.0298 (7)	−0.0047 (6)
S2	0.0449 (8)	0.0520 (8)	0.0570 (8)	−0.0081 (6)	−0.0146 (6)	−0.0037 (6)
P1	0.0431 (7)	0.0367 (7)	0.0380 (7)	−0.0127 (6)	−0.0100 (5)	−0.0038 (5)
P2	0.0466 (8)	0.0403 (7)	0.0475 (8)	−0.0142 (6)	−0.0124 (6)	−0.0046 (6)
C1	0.043 (3)	0.043 (3)	0.037 (3)	−0.012 (2)	−0.003 (2)	−0.001 (2)
C2	0.046 (3)	0.042 (3)	0.043 (3)	−0.007 (2)	−0.010 (2)	0.000 (2)
C3	0.063 (4)	0.061 (4)	0.063 (4)	−0.014 (3)	−0.026 (3)	0.002 (3)
C4	0.070 (4)	0.051 (4)	0.079 (4)	−0.003 (3)	−0.023 (3)	0.020 (3)
C5	0.073 (4)	0.037 (3)	0.092 (5)	−0.011 (3)	−0.024 (4)	0.015 (3)
C6	0.061 (3)	0.042 (3)	0.064 (3)	−0.019 (3)	−0.017 (3)	0.003 (3)
C7	0.045 (3)	0.038 (3)	0.039 (3)	−0.018 (2)	−0.006 (2)	−0.003 (2)
C8	0.054 (3)	0.048 (3)	0.048 (3)	−0.017 (3)	−0.008 (2)	−0.005 (2)
C9	0.061 (4)	0.065 (4)	0.052 (3)	−0.024 (3)	0.011 (3)	−0.019 (3)
C10	0.100 (5)	0.080 (4)	0.039 (3)	−0.035 (4)	−0.009 (3)	−0.012 (3)
C11	0.094 (5)	0.080 (4)	0.048 (3)	−0.028 (4)	−0.027 (3)	−0.002 (3)
C12	0.053 (3)	0.059 (3)	0.051 (3)	−0.015 (3)	−0.014 (3)	−0.011 (3)
C13	0.048 (3)	0.048 (3)	0.039 (3)	−0.014 (2)	−0.008 (2)	0.000 (2)
C14	0.072 (4)	0.076 (4)	0.064 (4)	−0.039 (3)	−0.006 (3)	−0.006 (3)
C15	0.076 (5)	0.101 (5)	0.085 (5)	−0.054 (4)	−0.006 (4)	−0.002 (4)
C16	0.055 (4)	0.103 (5)	0.081 (5)	−0.031 (4)	0.003 (3)	0.007 (4)
C17	0.058 (4)	0.076 (4)	0.064 (4)	−0.008 (3)	0.007 (3)	0.001 (3)
C18	0.057 (3)	0.051 (3)	0.056 (3)	−0.010 (3)	−0.010 (3)	−0.004 (3)
C19	0.073 (4)	0.042 (3)	0.046 (3)	−0.020 (3)	−0.020 (3)	−0.005 (2)
C20	0.068 (4)	0.055 (4)	0.094 (5)	−0.021 (3)	−0.028 (4)	0.012 (3)
C21	0.100 (5)	0.056 (4)	0.106 (6)	−0.015 (4)	−0.054 (5)	0.011 (4)
C22	0.136 (7)	0.056 (4)	0.072 (5)	−0.014 (5)	−0.030 (5)	0.006 (3)
C23	0.124 (7)	0.072 (5)	0.071 (5)	−0.033 (5)	0.002 (4)	0.012 (4)
C24	0.076 (4)	0.059 (4)	0.061 (4)	−0.011 (3)	−0.005 (3)	−0.001 (3)
C25	0.044 (3)	0.037 (3)	0.052 (3)	−0.013 (2)	−0.010 (2)	0.003 (2)
C26	0.056 (4)	0.074 (4)	0.064 (4)	−0.021 (3)	−0.008 (3)	−0.024 (3)
C27	0.058 (4)	0.071 (4)	0.074 (4)	−0.014 (3)	0.006 (3)	−0.013 (3)
C28	0.054 (4)	0.072 (4)	0.084 (4)	−0.016 (3)	−0.025 (3)	0.001 (4)
C29	0.056 (4)	0.083 (4)	0.067 (4)	−0.023 (3)	−0.022 (3)	−0.012 (3)
C30	0.049 (3)	0.054 (3)	0.046 (3)	−0.014 (3)	−0.012 (2)	−0.006 (2)
C31	0.039 (3)	0.044 (3)	0.058 (3)	−0.011 (2)	−0.010 (2)	−0.011 (3)
C32	0.063 (4)	0.053 (3)	0.055 (3)	−0.016 (3)	−0.001 (3)	−0.004 (3)
C33	0.092 (5)	0.088 (5)	0.055 (4)	−0.035 (4)	0.001 (3)	−0.016 (3)
C34	0.057 (4)	0.105 (6)	0.076 (5)	−0.032 (4)	0.008 (3)	−0.044 (4)
C35	0.064 (4)	0.060 (4)	0.100 (5)	−0.014 (3)	−0.006 (4)	−0.040 (4)
C36	0.054 (3)	0.054 (3)	0.069 (4)	−0.017 (3)	−0.012 (3)	−0.015 (3)
C38	0.053 (2)	0.056 (3)	0.063 (3)	−0.012 (2)	−0.024 (2)	−0.001 (2)
N37	0.056 (3)	0.068 (6)	0.080 (4)	−0.014 (4)	−0.015 (3)	0.005 (4)
C39	0.055 (4)	0.077 (6)	0.060 (4)	−0.011 (4)	−0.021 (3)	0.003 (4)
C40	0.066 (5)	0.089 (6)	0.063 (4)	−0.019 (4)	−0.025 (4)	0.008 (4)
C41	0.066 (5)	0.092 (6)	0.071 (4)	−0.022 (4)	−0.012 (4)	0.001 (4)
C42	0.061 (4)	0.078 (6)	0.082 (5)	−0.011 (4)	−0.008 (3)	0.003 (5)
N37A	0.054 (3)	0.067 (6)	0.081 (4)	−0.017 (4)	−0.018 (3)	0.007 (5)
C39A	0.055 (4)	0.077 (6)	0.060 (4)	−0.013 (4)	−0.022 (3)	0.007 (4)
C40A	0.064 (4)	0.091 (6)	0.066 (3)	−0.018 (4)	−0.024 (4)	0.010 (4)
C41A	0.067 (5)	0.091 (6)	0.070 (4)	−0.020 (5)	−0.014 (4)	0.003 (4)
C42A	0.055 (4)	0.077 (6)	0.081 (4)	−0.016 (4)	−0.012 (3)	0.001 (5)

### Geometric parameters (Å, °)

**Table:**

Pd1—P1	2.2585 (15)	C21—H21	0.9300
Pd1—P2	2.3575 (15)	C22—C23	1.359 (9)
Pd1—S1	2.2999 (15)	C22—H22	0.9300
Pd1—S2	2.3374 (15)	C23—C24	1.387 (8)
S1—C2	1.764 (5)	C23—H23	0.9300
S2—C38	1.735 (6)	C24—H24	0.9300
P1—C1	1.815 (5)	C25—C30	1.382 (6)
P1—C7	1.819 (5)	C25—C26	1.384 (7)
P1—C13	1.821 (5)	C26—C27	1.382 (8)
P2—C25	1.826 (5)	C26—H26	0.9300
P2—C31	1.827 (5)	C27—C28	1.362 (8)
P2—C19	1.828 (5)	C27—H27	0.9300
C1—C2	1.380 (6)	C28—C29	1.375 (8)
C1—C6	1.394 (6)	C28—H28	0.9300
C2—C3	1.394 (7)	C29—C30	1.359 (7)
C3—C4	1.362 (8)	C29—H29	0.9300
C3—H3	0.9300	C30—H30	0.9300
C4—C5	1.371 (8)	C31—C36	1.383 (7)
C4—H4	0.9300	C31—C32	1.384 (7)
C5—C6	1.368 (7)	C32—C33	1.382 (7)
C5—H5	0.9300	C32—H32	0.9300
C6—H6	0.9300	C33—C34	1.355 (9)
C7—C12	1.379 (6)	C33—H33	0.9300
C7—C8	1.384 (6)	C34—C35	1.362 (9)
C8—C9	1.378 (7)	C34—H34	0.9300
C8—H8	0.9300	C35—C36	1.366 (8)
C9—C10	1.358 (8)	C35—H35	0.9300
C9—H9	0.9300	C36—H36	0.9300
C10—C11	1.365 (8)	C38—N37	1.362 (8)
C10—H10	0.9300	C38—N37A	1.365 (8)
C11—C12	1.393 (7)	C38—C39A	1.384 (9)
C11—H11	0.9300	C38—C39	1.385 (9)
C12—H12	0.9300	N37—C42	1.363 (9)
C13—C18	1.372 (7)	C39—C40	1.339 (10)
C13—C14	1.383 (7)	C39—H39	0.9300
C14—C15	1.385 (8)	C40—C41	1.351 (10)
C14—H14	0.9300	C40—H40	0.9300
C15—C16	1.355 (9)	C41—C42	1.351 (11)
C15—H15	0.9300	C41—H41	0.9300
C16—C17	1.353 (9)	C42—H42	0.9300
C16—H16	0.9300	N37A—C42A	1.362 (9)
C17—C18	1.383 (7)	C39A—C40A	1.336 (10)
C17—H17	0.9300	C39A—H39A	0.9300
C18—H18	0.9300	C40A—C41A	1.346 (10)
C19—C24	1.377 (7)	C40A—H40A	0.9300
C19—C20	1.377 (7)	C41A—C42A	1.350 (11)
C20—C21	1.376 (8)	C41A—H41A	0.9300
C20—H20	0.9300	C42A—H42A	0.9300
C21—C22	1.348 (9)		
			
P1—Pd1—S1	87.50 (5)	C22—C21—H21	120.8
P1—Pd1—S2	87.20 (5)	C20—C21—H21	120.8
S1—Pd1—S2	174.33 (5)	C21—C22—C23	120.8 (7)
P1—Pd1—P2	178.22 (5)	C21—C22—H22	119.6
S1—Pd1—P2	91.42 (5)	C23—C22—H22	119.6
S2—Pd1—P2	93.83 (5)	C22—C23—C24	120.7 (7)
C2—S1—Pd1	105.60 (16)	C22—C23—H23	119.6
C38—S2—Pd1	102.20 (18)	C24—C23—H23	119.6
C1—P1—C7	106.4 (2)	C19—C24—C23	119.7 (6)
C1—P1—C13	106.3 (2)	C19—C24—H24	120.1
C7—P1—C13	106.1 (2)	C23—C24—H24	120.1
C1—P1—Pd1	107.42 (16)	C30—C25—C26	118.4 (5)
C7—P1—Pd1	114.19 (15)	C30—C25—P2	123.4 (4)
C13—P1—Pd1	115.84 (16)	C26—C25—P2	118.2 (4)
C25—P2—C31	102.7 (2)	C27—C26—C25	119.8 (5)
C25—P2—C19	103.6 (2)	C27—C26—H26	120.1
C31—P2—C19	104.2 (2)	C25—C26—H26	120.1
C25—P2—Pd1	114.30 (16)	C28—C27—C26	121.1 (6)
C31—P2—Pd1	110.55 (16)	C28—C27—H27	119.5
C19—P2—Pd1	119.64 (16)	C26—C27—H27	119.5
C2—C1—C6	120.2 (4)	C27—C28—C29	119.0 (5)
C2—C1—P1	117.0 (4)	C27—C28—H28	120.5
C6—C1—P1	122.8 (4)	C29—C28—H28	120.5
C1—C2—C3	119.3 (5)	C30—C29—C28	120.7 (5)
C1—C2—S1	122.4 (4)	C30—C29—H29	119.6
C3—C2—S1	118.3 (4)	C28—C29—H29	119.6
C4—C3—C2	119.5 (5)	C29—C30—C25	121.0 (5)
C4—C3—H3	120.3	C29—C30—H30	119.5
C2—C3—H3	120.3	C25—C30—H30	119.5
C3—C4—C5	121.6 (5)	C36—C31—C32	118.3 (5)
C3—C4—H4	119.2	C36—C31—P2	123.6 (4)
C5—C4—H4	119.2	C32—C31—P2	118.1 (4)
C6—C5—C4	119.7 (5)	C33—C32—C31	119.8 (5)
C6—C5—H5	120.1	C33—C32—H32	120.1
C4—C5—H5	120.1	C31—C32—H32	120.1
C5—C6—C1	119.7 (5)	C34—C33—C32	120.6 (6)
C5—C6—H6	120.1	C34—C33—H33	119.7
C1—C6—H6	120.1	C32—C33—H33	119.7
C12—C7—C8	118.5 (4)	C33—C34—C35	120.3 (6)
C12—C7—P1	121.5 (4)	C33—C34—H34	119.9
C8—C7—P1	119.5 (4)	C35—C34—H34	119.9
C9—C8—C7	121.0 (5)	C34—C35—C36	119.9 (6)
C9—C8—H8	119.5	C34—C35—H35	120.1
C7—C8—H8	119.5	C36—C35—H35	120.1
C10—C9—C8	120.1 (5)	C35—C36—C31	121.2 (6)
C10—C9—H9	119.9	C35—C36—H36	119.4
C8—C9—H9	119.9	C31—C36—H36	119.4
C9—C10—C11	120.0 (5)	N37—C38—C39A	126.8 (8)
C9—C10—H10	120.0	N37A—C38—C39A	117.8 (7)
C11—C10—H10	120.0	N37—C38—C39	118.1 (7)
C10—C11—C12	120.4 (6)	N37A—C38—C39	115.0 (8)
C10—C11—H11	119.8	N37—C38—S2	117.8 (6)
C12—C11—H11	119.8	N37A—C38—S2	124.1 (5)
C7—C12—C11	119.9 (5)	C39A—C38—S2	115.3 (6)
C7—C12—H12	120.0	C39—C38—S2	120.7 (6)
C11—C12—H12	120.0	C38—N37—C42	116.3 (7)
C18—C13—C14	118.0 (5)	C40—C39—C38	121.1 (8)
C18—C13—P1	119.7 (4)	C40—C39—H39	119.4
C14—C13—P1	122.3 (4)	C38—C39—H39	119.4
C13—C14—C15	120.1 (6)	C39—C40—C41	119.9 (8)
C13—C14—H14	119.9	C39—C40—H40	120.1
C15—C14—H14	119.9	C41—C40—H40	120.0
C16—C15—C14	120.7 (6)	C42—C41—C40	117.6 (8)
C16—C15—H15	119.6	C42—C41—H41	121.2
C14—C15—H15	119.6	C40—C41—H41	121.2
C17—C16—C15	119.9 (6)	C41—C42—N37	124.0 (8)
C17—C16—H16	120.0	C41—C42—H42	118.0
C15—C16—H16	120.0	N37—C42—H42	118.0
C16—C17—C18	120.0 (6)	C42A—N37A—C38	117.1 (7)
C16—C17—H17	120.0	C40A—C39A—C38	121.1 (8)
C18—C17—H17	120.0	C40A—C39A—H39A	119.5
C13—C18—C17	121.2 (5)	C38—C39A—H39A	119.5
C13—C18—H18	119.4	C39A—C40A—C41A	120.6 (8)
C17—C18—H18	119.4	C39A—C40A—H40A	119.7
C24—C19—C20	117.4 (5)	C41A—C40A—H40A	119.7
C24—C19—P2	122.5 (4)	C40A—C41A—C42A	117.3 (8)
C20—C19—P2	120.1 (4)	C40A—C41A—H41A	121.4
C21—C20—C19	122.8 (6)	C42A—C41A—H41A	121.4
C21—C20—H20	118.6	C41A—C42A—N37A	123.9 (8)
C19—C20—H20	118.6	C41A—C42A—H42A	118.1
C22—C21—C20	118.4 (7)	N37A—C42A—H42A	118.1
			
P1—Pd1—S1—C2	0.08 (17)	C31—P2—C19—C20	−56.4 (5)
P2—Pd1—S1—C2	178.66 (17)	Pd1—P2—C19—C20	67.8 (5)
P1—Pd1—S2—C38	−99.05 (19)	C24—C19—C20—C21	−3.4 (9)
P2—Pd1—S2—C38	82.41 (19)	P2—C19—C20—C21	178.3 (5)
S1—Pd1—P1—C1	−1.59 (15)	C19—C20—C21—C22	2.1 (10)
S2—Pd1—P1—C1	−179.60 (15)	C20—C21—C22—C23	1.0 (11)
S1—Pd1—P1—C7	−119.32 (18)	C21—C22—C23—C24	−2.5 (11)
S2—Pd1—P1—C7	62.67 (17)	C20—C19—C24—C23	1.8 (8)
S1—Pd1—P1—C13	116.98 (18)	P2—C19—C24—C23	−179.9 (5)
S2—Pd1—P1—C13	−61.03 (18)	C22—C23—C24—C19	1.1 (10)
S1—Pd1—P2—C25	29.13 (18)	C31—P2—C25—C30	−16.2 (5)
S2—Pd1—P2—C25	−153.00 (18)	C19—P2—C25—C30	92.1 (4)
S1—Pd1—P2—C31	−86.20 (18)	Pd1—P2—C25—C30	−136.0 (4)
S2—Pd1—P2—C31	91.67 (18)	C31—P2—C25—C26	164.9 (4)
S1—Pd1—P2—C19	152.8 (2)	C19—P2—C25—C26	−86.8 (4)
S2—Pd1—P2—C19	−29.3 (2)	Pd1—P2—C25—C26	45.1 (4)
C7—P1—C1—C2	126.1 (4)	C30—C25—C26—C27	−1.1 (8)
C13—P1—C1—C2	−121.2 (4)	P2—C25—C26—C27	177.9 (4)
Pd1—P1—C1—C2	3.4 (4)	C25—C26—C27—C28	0.9 (9)
C7—P1—C1—C6	−55.5 (4)	C26—C27—C28—C29	−0.6 (10)
C13—P1—C1—C6	57.2 (4)	C27—C28—C29—C30	0.5 (9)
Pd1—P1—C1—C6	−178.2 (4)	C28—C29—C30—C25	−0.8 (9)
C6—C1—C2—C3	−2.4 (7)	C26—C25—C30—C29	1.0 (8)
P1—C1—C2—C3	176.1 (4)	P2—C25—C30—C29	−177.9 (4)
C6—C1—C2—S1	177.7 (4)	C25—P2—C31—C36	95.0 (5)
P1—C1—C2—S1	−3.8 (5)	C19—P2—C31—C36	−12.8 (5)
Pd1—S1—C2—C1	2.2 (4)	Pd1—P2—C31—C36	−142.6 (4)
Pd1—S1—C2—C3	−177.7 (4)	C25—P2—C31—C32	−81.7 (4)
C1—C2—C3—C4	1.6 (8)	C19—P2—C31—C32	170.5 (4)
S1—C2—C3—C4	−178.5 (4)	Pd1—P2—C31—C32	40.7 (4)
C2—C3—C4—C5	0.2 (9)	C36—C31—C32—C33	−1.4 (8)
C3—C4—C5—C6	−1.3 (9)	P2—C31—C32—C33	175.5 (4)
C4—C5—C6—C1	0.6 (9)	C31—C32—C33—C34	2.2 (9)
C2—C1—C6—C5	1.3 (8)	C32—C33—C34—C35	−1.2 (10)
P1—C1—C6—C5	−177.1 (4)	C33—C34—C35—C36	−0.6 (9)
C1—P1—C7—C12	154.0 (4)	C34—C35—C36—C31	1.3 (9)
C13—P1—C7—C12	41.1 (5)	C32—C31—C36—C35	−0.3 (8)
Pd1—P1—C7—C12	−87.7 (4)	P2—C31—C36—C35	−177.0 (4)
C1—P1—C7—C8	−34.1 (4)	Pd1—S2—C38—N37	−1.2 (12)
C13—P1—C7—C8	−147.0 (4)	Pd1—S2—C38—N37A	15.2 (13)
Pd1—P1—C7—C8	84.2 (4)	Pd1—S2—C38—C39A	175.7 (9)
C12—C7—C8—C9	0.1 (7)	Pd1—S2—C38—C39	−159.9 (10)
P1—C7—C8—C9	−172.0 (4)	N37A—C38—N37—C42	64 (3)
C7—C8—C9—C10	−0.4 (8)	C39A—C38—N37—C42	5(3)
C8—C9—C10—C11	−0.6 (9)	C39—C38—N37—C42	−19 (2)
C9—C10—C11—C12	1.8 (9)	S2—C38—N37—C42	−178.3 (12)
C8—C7—C12—C11	1.0 (7)	N37—C38—C39—C40	17 (2)
P1—C7—C12—C11	173.0 (4)	N37A—C38—C39—C40	0(2)
C10—C11—C12—C7	−2.1 (9)	C39A—C38—C39—C40	−102 (2)
C1—P1—C13—C18	102.0 (4)	S2—C38—C39—C40	175.9 (10)
C7—P1—C13—C18	−145.1 (4)	C38—C39—C40—C41	−3(2)
Pd1—P1—C13—C18	−17.2 (5)	C39—C40—C41—C42	−8(2)
C1—P1—C13—C14	−75.3 (5)	C40—C41—C42—N37	6(2)
C7—P1—C13—C14	37.6 (5)	C38—N37—C42—C41	8(3)
Pd1—P1—C13—C14	165.5 (4)	N37—C38—N37A—C42A	−115 (5)
C18—C13—C14—C15	0.0 (8)	C39A—C38—N37A—C42A	15 (2)
P1—C13—C14—C15	177.4 (5)	C39—C38—N37A—C42A	−10 (2)
C13—C14—C15—C16	−0.1 (10)	S2—C38—N37A—C42A	174.8 (11)
C14—C15—C16—C17	0.2 (11)	N37—C38—C39A—C40A	−1(2)
C15—C16—C17—C18	−0.2 (10)	N37A—C38—C39A—C40A	−16 (2)
C14—C13—C18—C17	0.0 (8)	C39—C38—C39A—C40A	72.5 (19)
P1—C13—C18—C17	−177.4 (4)	S2—C38—C39A—C40A	−177.8 (10)
C16—C17—C18—C13	0.1 (9)	C38—C39A—C40A—C41A	5(2)
C25—P2—C19—C24	18.2 (5)	C39A—C40A—C41A—C42A	8(2)
C31—P2—C19—C24	125.4 (5)	C40A—C41A—C42A—N37A	−9(2)
Pd1—P2—C19—C24	−110.5 (4)	C38—N37A—C42A—C41A	−3(3)
C25—P2—C19—C20	−163.6 (4)		

### Table 2 Intra- and intermolecular C—H···π and π–π interactions (Å)

**Table:**

H/centroid	centroid	distance
N37,C38–C42	C19–C24	3.93 (2)
N37A,C38,C39A–C42A	C7–C12	4.00 (2)
C31–C36	C31^i^–C36^i^	3.76 (2)
H24	C25–C30	3.17
H30	C31–C36	3.11
H36	C19–C24	3.20
H29	C31^ii^–C36^ii^	3.06

Symmetry codes: (i) -x, -y+1, -z+1; (ii) -x+1, -y+1, -z+1.
